# The malaria testing and treatment landscape in Kenya: results from a nationally representative survey among the public and private sector in 2016

**DOI:** 10.1186/s12936-017-2089-0

**Published:** 2017-12-21

**Authors:** ACTwatch Group, Louis Akulayi, Angela Alum, Andrew Andrada, Julie Archer, Ekundayo D. Arogundade, Erick Auko, Abdul R. Badru, Katie Bates, Paul Bouanchaud, Meghan Bruce, Katia Bruxvoort, Peter Buyungo, Angela Camilleri, Emily D. Carter, Steven Chapman, Nikki Charman, Desmond Chavasse, Robyn Cyr, Kevin Duff, Gylsain Guedegbe, Keith Esch, Illah Evance, Anna Fulton, Hellen Gataaka, Tarryn Haslam, Emily Harris, Christine Hong, Catharine Hurley, Whitney Isenhower, Enid Kaabunga, Baraka D. Kaaya, Esther Kabui, Beth Kangwana, Lason Kapata, Henry Kaula, Gloria Kigo, Irene Kyomuhangi, Aliza Lailari, Sandra LeFevre, Megan Littrell, Greta Martin, Daniel Michael, Erik Monroe, Godefroid Mpanya, Felton Mpasela, Felix Mulama, Anne Musuva, Julius Ngigi, Edward Ngoma, Marjorie Norman, Bernard Nyauchi, Kathryn A. O’Connell, Carolyne Ochieng, Edna Ogada, Linda Ongwenyi, Ricki Orford, Saysana Phanalasy, Stephen Poyer, Justin Rahariniaina, Jacky Raharinjatovo, Lanto Razafindralambo, Solofo Razakamiadana, Christina Riley, John Rodgers, Andria Rusk, Tanya Shewchuk, Simon Sensalire, Julianna Smith, Phok Sochea, Tsione Solomon, Raymond Sudoi, Martine Esther Tassiba, Katherine Thanel, Rachel Thompson, Mitsuru Toda, Chinazo Ujuju, Marie-Alix Valensi, Vamsi Vasireddy, Cynthia B. Whitman, Cyprien Zinsou, Anne Musuva, Waqo Ejersa, Rebecca Kiptui, Dorothy Memusi, Edward Abwao

**Affiliations:** 1 Population Services International, 1120 19th St NW Suite 600, Washington, DC 20036 USA; 2 Population Services Kenya (PS/Kenya), 2nd Floor, Wing B, Jumuia Place, Lenana Road, P.O. Box 22591-00400, Nairobi, Kenya; 3 National Malaria Control Programme, Kenya KNH Grounds, P.O. Box 20750, Nairobi, Kenya; 4 Pharmacy and Poisons Board, Nairobi, Kenya

**Keywords:** Malaria control case management, Anti-malarial, ACT, Private sector, Public sector, Malaria diagnosis, Kenya

## Abstract

**Background:**

Since 2004, Kenya’s national malaria treatment guidelines have stipulated artemisinin-based combination therapy (ACT) as first-line treatment for uncomplicated malaria, and since 2014, confirmatory diagnosis of malaria in all cases before treatment has been recommended. A number of strategies to support national guidelines have been implemented in the public and private sectors in recent years. A nationally-representative malaria outlet survey, implemented across four epidemiological zones, was conducted between June and August 2016 to provide practical evidence to inform strategies and policies in Kenya towards achieving national malaria control goals.

**Results:**

A total of 17,852 outlets were screened and 2271 outlets were eligible and interviewed. 78.3% of all screened public health facilities stocked both malaria diagnostic testing and quality-assured ACT (QAACT). Sulfadoxine–pyrimethamine (SP) for intermittent preventive treatment in pregnancy was available in 70% of public health facilities in endemic areas where it is recommended for treatment. SP was rarely found in the public sector outside of the endemic areas (< 0.5%). The anti-malaria stocking private sector had lower levels of QAACT (46.7%) and malaria blood testing (20.8%) availability but accounted for majority of anti-malarial distribution (70.6% of the national market share). More than 40% of anti-malarials were distributed by unregistered pharmacies (37.3%) and general retailers (7.1%). QAACT accounted for 58.2% of the total anti-malarial market share, while market share for non-QAACT was 15.8% and for SP, 24.8%. In endemic areas, 74.9% of anti-malarials distributed were QAACT. Elsewhere, QAACT market share was 49.4% in the endemic-prone areas, 33.2% in seasonal-transmission areas and 37.9% in low-risk areas.

**Conclusion:**

Although public sector availability of QAACT and malaria diagnosis is relatively high, there is a gap in availability of both testing and treatment that must be addressed. The private sector in Kenya, where the majority of anti-malarials are distributed, is also critical for achieving universal coverage with appropriate malaria case management. There is need for a renewed commitment and effective strategies to ensure access to affordable QAACT and confirmatory testing in the private sector, and should consider how to address malaria case management among informal providers responsible for a substantial proportion of the anti-malarial market share.

**Electronic supplementary material:**

The online version of this article (10.1186/s12936-017-2089-0) contains supplementary material, which is available to authorized users.

## Background

Malaria is the leading cause of morbidity and mortality in Kenya, with over 70% of the population at risk of infection [1]. In 2013, there were over 2.3 million confirmed cases of malaria, accounting for more than 20% of outpatient visits, 19% of hospital admissions and 3–5% of hospital deaths [2]. However, there have been important reductions in malaria prevalence in recent years. Between 2010 and 2015, national data reveal malaria prevalence in children aged 6 months to 14 years fell from 11 to 8% respectively. Furthermore, from 2011 to 2015 the suspected outpatient malaria cases, as a proportion of the outpatient department cases, declined from over 35 to 15% [3]. However, regional variations are apparent, and analysis has shown a trend of increased prevalence of *Plasmodium falciparum* in the coastal-endemic area over the same period [3, 4].

Given the variability in malaria transmission throughout the country, the Kenya National Malaria Control Programme has defined epidemiological zones based on malaria risk and burden [5]. These malaria epidemiological zones are largely defined by altitude, rainfall patterns and temperature. They include: endemic areas around Lake Victoria in western Kenya and in the coastal region; highland epidemic-prone areas of western Kenya and the Rift Valley region; seasonal-transmission areas, which are the arid and semi-arid areas of the northern and south eastern parts of the country; and low-risk areas in the central highlands of Kenya including Nairobi [5]. These epidemiological zones are used to guide the implementation of malaria control interventions, including intermittent preventive treatment in pregnancy (IPTp) and community case management, which are both focused in endemic areas.

Following a recognition that sulfadoxine–pyrimethamine (SP) was failing, in 2004 the National Malaria Control Programme adopted artemether–lumefantrine (AL) as the first-line treatment for uncomplicated malaria, with the change being widely implemented from 2006 onwards [6]. The national malaria control guidelines, recommend dihydroartemisinin-piperaquine as the second-line treatment for uncomplicated malaria in Kenya. The treatment recommendation for severe malaria is parenteral artesunate, while treatment with parenteral quinine is permitted in absence of artesunate. Patients with severe malaria should be referred to higher levels of care. SP is recommended for IPTp only in the malaria endemic zones. The 2014 Kenya national malaria control guidelines recommend confirmatory diagnosis of malaria in all age-groups of patients in all epidemiological settings [7]. This was a departure from the previous guidelines that recommended presumptive treatment.

## National malaria control strategies and interventions for case management

Several strategies have been implemented to ensure access to quality case management services and commodities. Between 2010 and 2013, Kenya and other six countries (Ghana, Madagascar, Niger, Nigeria, Tanzania [including Zanzibar] and Uganda) participated in the Affordable Medicines Facility for malaria (AMFm). The AMFm provided quality-assured ACT (QAACT) to wholesalers at a heavily-subsidized cost with the objective of increasing access to affordable ACT in both the public and private sector [8]. The packaging of AMFm-subsidized QAACT was marked with a distinctive green leaf logo for easy identification. By the end of 2011, approximately 14.35 million co-paid QAACT treatments were delivered to Kenya’s public sector and 14.1 million to the private sector [9]. The AMFm independent evaluation reported significant improvements in availability, price, and relative market share of QAACT in Kenya, and especially in the private sector [8, 10].

Building on the successes of the AMFm, the Global Fund introduced a new funding model, known as the co-payment mechanism (CPM), to support private sector access to QAACT medicines. The CPM focused exclusively on the private sector supply of QAACT given that the independent evaluation showed that the AMFm had greater impact on the supply of QAACT in the private than compared to the public sector [10]. However, the public sector continued to receive subsidized ACT through an alternative Global Fund mechanism. During the CPM, QAACTs in this sector were not marked with the green leaf logo. While substantial resources were provided by donor communities for procurement of subsidized QAACT in Kenya for the period between 2013 and 2015, resources were not at their peak as during the AMFm period. In 2015, only 6.85 million treatments were delivered to the private sector through the CPM. The ACT subsidy was also decreased to wholesalers from 90 to 70% for all pack sizes [9], lending to a recommended retail price of $1.00 to the consumer, for both children and adults. Finally, while several mass communication activities were implemented to increase demand and consumer awareness of QAACT, these were discontinued in mid-2015 [9].

In addition to the AMFm and CPM, other strategies have been implemented to increase availability and demand for rapid diagnostic tests (RDTs) [5, 11]. In the private sector, this was supported by changing national policy to allow point-of-service testing beyond private hospitals and large private for-profit health facilities to registered pharmacies. In the public sector, RDTs were permitted and supplied to lower-level facilities, including community health workers (CHW) operating in malaria endemic areas.

Data on the anti-malarial and malaria diagnostic markets in Kenya provide an important benchmark to measure the extent to which malaria case management services are available and are aligned with national malaria control guidelines. The ACTwatch project, a multi-country research project that was launched in 2008, provides timely, relevant and high quality evidence for this purpose [12]. The objective of this paper is to provide practical evidence to inform strategies and policies in Kenya towards achieving national malaria control goals, by describing the total market for malaria medicines and diagnostics at national level. This paper presents data from the recently conducted outlet survey in 2016.

## Methods

### Design

A nationally-representative, cross-sectional quantitative survey was conducted among outlets with potential to stock anti-malarials or malaria diagnosis. All potential public and private sector outlets were included in the survey. The public sector included all tiers of the health care system (hospitals, health centers, dispensaries, clinics and CHWs) owned by government or affiliated with the not-for profit organizations such as non-governmental and faith-based institutions. Outlets surveyed in the private sector included, private for-profit health facilities (hospitals, nursing homes/medical centers and clinics), pharmacies and chemists (registered and unregistered) and general retailers selling fast-moving consumer goods. Table 1 provides an additional description of the outlet types.

**Table 1:** Outlet descriptions

Public health facilities	Tertiary referral hospital, secondary referral hospitals, primary referral hospitals, health centers and dispensaries. There are over 3000 Ministry of Health public health facilities in Kenya
Community health worker	Community-based health workers, including community health volunteers
Private for-profit health facilities	Private hospitals, clinics, dispensaries, and diagnostic laboratories. There are over 3100 registered private for-profit health facilities in Kenya
Registered pharmacies/chemists	Pharmacies are licensed and regulated by the Pharmacy and Poisons Board and include Pharmacist Premises. By 2014, there were over 3900 registered Pharmacies in Kenya
Unregistered pharmacies/chemists	Small businesses that are not registered with the Pharmacy and Poisons Board, but sell various classes of prescription and over-the-counter medicine at commercial prices
General retailers	Supermarkets, duka, kiosks, market stalls, and petrol stations

### Sampling

The 2016 survey was stratified to deliver estimates for each of the aforementioned malaria epidemiological zones in Kenya. Clusters were selected from the four malaria epidemiological zones and defined as (1) endemic areas, (2) epidemic-prone areas, (3) seasonal-transmission areas, and (4) low-risk areas. Considering that updated and comprehensive lists of all potentially eligible outlets were not routinely available at both national and sub-national levels, a cluster sampling approach with an outlet census was used to identify outlets for inclusion. A cluster was defined as an administrative unit ideally with a population of 10,000–15,000 inhabitants and this corresponded with a “location”. Using 2009 Kenya Population and Housing Census [13], a national sampling framework was constructed and survey clusters or locations were selected using the technique of probability proportional to population size.

The survey was powered to detect a minimum of a 10-percentage point change in availability of QAACT medicines within each strata at the 5% significance level with 80% power. The number of study clusters was calculated for each strata based on the required number of anti-malarial stocking outlets, assumptions about the number of anti-malarial stocking outlets per cluster and information from previous survey rounds including anti-malarial and QAACT availability, outlet density per cluster, and design effect. A total of 84 locations were sampled, this included 17 endemic locations, 22 epidemic-prone locations, 28 seasonal-transmission locations, and 17 low-risk locations. Within each sampled location, all outlets with the potential to provide anti-malarials or diagnostic testing services to patients or clients were screened for eligibility. In all sampled locations, the census boundary was extended to the higher administrative unit, the “division”, to allow for over sampling of public health facilities that are relatively uncommon at the location level but important outlet type in health service provision.

### Training and fieldwork

Data were collected between 7th June and 17th August, 2016 by 14 data collection teams. All fieldworkers attended a standardized training that consisted of classroom presentations, exercises and role plays as well as a field exercise. Additional training was provided for supervisors and quality-controllers that focused on field monitoring, verification visits, and census procedures. Data collection teams were provided with a list of sampled locations and official maps that illustrated their administrative boundaries. In each sampled location, fieldworkers conducted a systematic and full enumeration of all outlets.

### Measures

Data were collected using a standardized ACTwatch outlet survey questionnaire and key informant interviews. Using the outlet survey questionnaire, the primary provider/owner of each potential outlet was invited to participate in the study and screening questions were administered to assess eligibility. Consenting providers were asked to show the interviewer all anti-malarials and malaria RDTs currently available. An anti-malarial audit sheet was completed to capture information for each unique anti-malarial product in the outlet, including formulation, brand name, active ingredients and strengths, package size, manufacturer and country of manufacture. Providers were asked to report the retail and wholesale cost for each medicine, as well as the amount distributed to individual consumers in the last week. Similarly, among the outlets found stocking malaria RDTs, an audit was completed to record information such as brand name, manufacturer, country of manufacturer, reported retail selling price and number of tests conducted or sold in the last 7 days for each of unique RDT product. Finally, a provider module was administered to assess provider’s knowledge and reported practices on malaria case-management policy recommendations. Outlet survey data were captured using Android phones fitted with customized forms created using DroidDB (© SYWARE, Inc., Cambridge, MA, USA). Interviews were conducted in local language using questionnaires that were translated from English to Swahili language and back to English to confirm translations.

### Protection of human subjects

The outlet survey protocol received ethical approval from Kenyatta National Hospital–University of Nairobi Ethics & Research Committee (Reference Number KNH-ERC/A/145). Provider interviews and product audits were completed only after administration of a standard informed consent form and provider consent to participate in the study. Standard measures were employed to maintain respondent confidentiality and anonymity, such as ensuring privacy during interviews, secure storage of completed questionnaires, and preventing any sharing of data between outlets. Providers had the option to end the interview at any point during the study.

### Data analysis

Data were analysed using Stata version 13.1 (StataCorp College Station, Texas, USA). Standard indicators were constructed according to definitions applied across the ACTwatch project and have been described elsewhere [12, 14]. Descriptive analysis was undertaken, all point estimates and 95% confidence intervals were weighted to provide national estimates and calculated using Stata survey setting procedures to account for the complex clustered and stratified sampling strategy. The sampling weights were calculated as the inverse of the probability of cluster selection. Data were presented according to the four strata as well as by outlet types.

### Definitions

According to information on drug formulation, active pharmaceutical ingredients and strengths, anti-malarials were classified as non-artemisinin therapies, artemisinin monotherapies and ACTs. ACT medicines were further classified as either QAACT or non-quality-assured ACT (non-QAACT). QAACT was defined as ACT medicines that had World Health Organization prequalification status, ACT medicines in compliance with the Global Fund quality assurance policy, or ACT medicines granted regulatory approval by the European Medicines Agency. ACT medicines that did not meet requirements for QAACTs were categorized as non-QAACTs.

Availability of any anti-malarial was calculated with all screened outlets as the denominator. In the public sector, the availability of specific types of anti-malarials was calculated using the denominator of all screened outlets given that anti-malarials should be available at all public health facilities and among CHWs. Availability of specific anti-malarial categories in the private sector was calculated using the total number of private sector outlets stocking any anti-malarial as the denominator. Outlet “readiness” for malaria case management was defined as the extent to which an outlet had QAACT and malaria testing available.

This paper also presents market share and price indicators among different classes of anti-malarials. The measure of adult equivalent treatment dose (AETD) was deployed to analyse market share and price to allow for meaningful comparisons between anti-malarials with different treatment courses. The AETD was defined as the amount of active ingredient required to treat an adult weighing 60 kg according to World Health Organization treatment guidelines [15]. Provider reports on the amount of the drug sold or distributed during the week preceding the survey were used to calculate sales or distribution volume according to type of anti-malarial. All dosage formulations were included in the sales or distribution volume calculation to provide a complete assessment of anti-malarial market share to the consumer or patient. Volumes were therefore the number of AETDs sold or distributed by a provider in the 7 days prior to the survey. Additional public health facilities sampled as part of over-sampling for these outlet types were not included in market share calculations.

Price data were collected in Kenya Shillings and converted to United States Dollar based on official exchange rates for the data collection period. Anti-malarial price indicators are expressed as median unit cost for one AETD to allow for comparability between classes of anti-malarials. Only tablet formulations are reported due to the differences in unit costs for tablet and non-tablet formulations. The interquartile range (IQR) was calculated to demonstrate price dispersion.

Provider perceptions regarding the most effective first-line treatment was assessed by administering questions to the senior most provider at all anti-malarial-stocking outlets. Providers were asked to describe what medicine they believed was the most effective treatment for uncomplicated malaria in a child and in an adult.

## Results

### Sample size description

A total of 17,852 outlets were screened for availability of anti-malarials and/or malaria blood testing services. Of screened outlets, 2291 were stocking anti-malarials or testing on the day of the survey or within the past 3 months, and 2271 were subsequently interviewed. A total of 1917 eligible and interviewed outlets were stocking anti-malarials on the day of the survey, 293 reportedly stocked anti-malarial(s) within the past 3 months while 61 were found stocking malaria diagnostics without anti-malarial products. A total of 6716 anti-malarial medicines and 846 RDT products were audited (Additional file 1).

### Public sector availability

Table 2 summarizes the availability of anti-malarials and diagnostics among the screened public sector outlets on the day of survey. Availability of any anti-malarial medicine was 91.8 and 2.4% among CHW, and this was highest in the endemic areas (99.0%) and lowest in the low-risk areas (81.0%). Among the screened outlets, 87.1% of public health facilities had QAACT medicines available and this varied by epidemiological zone: endemic areas (92.2%), endemic-prone areas (93.5%), seasonal-transmission areas (87.5%) and low-risk areas (77.0%). Public health facility availability of weight specific QA AL was variable: 66.4% for 6 tablet-pack QA AL, 63.3% for 12 tablet-pack QA AL, 37.0% for 18 tablet-pack QA AL and 72.7% for adult tablet-pack of QA AL (Additional file 2). Availability of non-QAACT medicines among all screened public health facilities was 12.3% and highest in the low-risk areas (17.8%). Overall availability of SP among the screened public health facilities was 17.6%, but this varied by epidemiological zone, where availability was 70.0% in endemic areas and less than 1% across all other epidemiological zones. Of all screened public health facilities, 46.0% stocked artesunate injection and this varied by epidemiological zone: endemic areas (59.8%), endemic-prone areas (63.9%), seasonal-transmission areas (48.9%) and low-risk areas (16.0%).

**Table 2:** Availability of anti-malarials in the public sector, among all screened outlets

	Public Health Facility^a^	CHW^b^
	% (95% CI)	% (95% CI)
Any anti-malarial		
Total	91.8 (89.2, 93.8)	2.4 (0.9, 6.3)
Endemic	99.0 (95.8, 99.8)	4.2 (1.0, 15.6)
Endemic-prone	97.6 (91.9, 99.3)	3.5 (0.8, 14.9)
Low-risk	81.0 (72.2, 87.5)	0.0 (–)
Seasonal-transmission	91.5 (86.5, 94.7)	0.7 (0.2, 2.2)
QAACT		
Total	87.1 (83.0, 90.4)	2.4 (0.9, 6.3)
Endemic	92.2 (77.8, 97.6)	4.2 (1.0, 15.6)
Endemic-prone	93.5 (85.0, 97.3)	3.5 (0.8, 14.9)
Low-risk	77.0 (66.1, 85.2)	0.0 (–)
Seasonal-transmission	87.5 (80.1, 92.4)	0.7 (0.2, 2.2)
Non-QAACT		
Total	12.3 (7.9, 18.7)	0.1 (0.0, 0.3)
Endemic	9.3 (3.4, 22.9)	0.1 (0.0, 0.7)
Endemic-prone	10.7 (5.1, 20.8)	0.1 (0.0, 1.2)
Low-risk	17.8 (7.2, 37.9)	0.0 (–)
Seasonal-transmission	10.8 (4.2, 25.2)	0.0 (–)
SP		
Total	17.6 (13.7, 22.3)	0.0 (–)
Endemic	70.0 (56.4, 80.8)	0.0 (–)
Endemic-prone	0.4 (0.0, 3.2)	0.0 (–)
Low-risk	0.1 (0.0, 0.6)	0.0 (–)
Seasonal-transmission	0.0 (–)	0.0 (–)
Artesunate IV/IM		
Total	46.0 (39.7, 52.3)	0.0 (–)
Endemic	59.8 (41.6, 75.7)	0.0 (–)
Endemic-prone	63.9 (50.9, 75.2)	0.0 (–)
Low-risk	16.0 (6.8, 33.4)	0.0 (–)
Seasonal-transmission	48.9 (39.3, 58.7)	0.0 (–)

^a^Endemic, N = 182; Endemic-prone, N = 246; Low-risk, N = 151; Seasonal-transmission, N = 217

^b^Endemic, N = 682; Endemic-prone, N = 312; Low-risk, N = 316; Seasonal-transmission, N = 505

Of all screened public health facilities, 86.4% had capacity for malaria blood testing, more commonly through RDTs (69.7%) compared to malaria microscopy (44.2%) (Table 3). Malaria blooding testing was lowest among public health facilities in endemic areas (79.9%). Among all screened CHWs, availability of RDTs was 4.3% and was highest in the endemic areas (7.3%) and endemic-prone areas (8.0%), compared to the seasonal-transmission areas and low-risk areas where availability was less than 1%.

**Table 3:** Availability of malaria diagnostic testing in the public sector, among all screened outlets

	Public Health Facility^a^	CHW^b^
	% (95% CI)	% (95% CI)
Any diagnostic test		
Total	86.4 (82.4, 89.6)	4.3 (2.2, 8.2)
Endemic	79.9 (68.8, 87.8)	7.3 (3.1, 16.2)
Endemic-prone	89.9 (84.7, 93.4)	8.0 (2.2, 24.8)
Low-risk	88.4 (77.1, 94.5)	0.0 (–)
Seasonal-transmission	87.6 (82.5, 91.4)	0.4 (0.1, 1.6)
Microscopy		
Total	44.2 (40.1, 48.4)	0.1 (0.0, 0.4)
Endemic	48.3 (41.2, 55.5)	0.0 (–)
Endemic-prone	47.0 (39.0, 55.2)	0.0 (–)
Low-risk	45.7 (36.3, 55.5)	0.0 (–)
Seasonal-transmission	36.8 (28.4, 46.1)	0.2 (0.0, 1.6)
RDT		
Total	69.7 (62.8, 75.9)	4.3 (2.2, 8.2)
Endemic	57.3 (40.7, 72.4)	7.3 (3.1, 16.2)
Endemic-prone	69.9 (60.3, 78.1)	8.0 (2.2, 24.8)
Low-risk	80.5 (65.7, 89.8)	0.0 (–)
Seasonal-transmission	70.5 (57.9, 80.6)	0.4 (0.1, 1.6)

^a^Endemic, N = 182; Endemic-prone, N = 246; Low-risk, N = 151; Seasonal-transmission, N = 217

^b^Endemic, N = 682; Endemic-prone, N = 312; Low-risk, N = 316; Seasonal-transmission, N = 505

Among all screened public health facilities, 78.3% had both QAACT medicines and malaria blood testing available, and this varied by epidemiological zone: endemic areas (75.6%), endemic-prone areas (84.6%), seasonal-transmission areas (72.8%) and low-risk areas (81.4%) (Table 4). Only 8.8% of public health facilities had QAACT medicines available but no malaria blood testing, and this was highest in the endemic areas (16.7%).

**Table 4:** Readiness for malaria case management in the public sector, among all screened outlets

	Public Health Facility^a^	CHW^b^
	% (95% CI)	% (95% CI)
QAACT and malaria testing available		
Total	78.3 (73.6, 82.4)	1.9 (0.7, 4.9)
Endemic	75.6 (61.9, 85.5)	3.2 (0.8, 11.8)
Endemic-prone	84.6 (76.5, 90.3)	3.3 (0.7, 13.9)
Low-risk	72.8 (61.6, 81.7)	0.0 (-)
Seasonal-transmission	81.4 (73.1, 87.5)	0.4 (0.1, 1.6)
QAACT available but no malaria testing available		
Total	8.8 (6.2, 12.3)	0.5 (0.2, 1.7)
Endemic	16.7 (9.2, 28.3)	1.0 (0.2, 4.7)
Endemic-prone	8.8 (5.6, 13.7)	0.3 (0.0, 2.3)
Low-risk	4.2 (1.5, 11.7)	0.0 (-)
Seasonal-transmission	6.1 (3.4, 10.7)	0.3 (0.1, 1.8)

^a^Endemic, N = 182; Endemic-prone, N = 246; Low-risk, N = 151; Seasonal-transmission, N = 217

^b^Endemic, N = 682; Endemic-prone, N = 312; Low-risk, N = 316; Seasonal-transmission, N = 505

### Private sector availability

Among all screened private sector outlets, availability of any anti-malarial varied by facility type: private-for-profit facilities (76.4%), registered pharmacies (93.3%), un-registered pharmacies (87.2%), and general retailers (2.4%) (Table 5). Among the anti-malarial stocking private sector outlets 46.7% had QAACT medicines, and this was highest among registered pharmacies (73.2%) and lowest among general retailers (9.9%). Private sector availability of QAACT medicines also varied by epidemiological zone: endemic areas (66.7%), endemic-prone areas (43.0%), seasonal-transmission areas (33.8%) and low-risk areas (42.2%) (Additional file 3). Availability of non-QAACT medicines was 37.9% in the private sector, and highest among anti-malarial stocking registered pharmacies (76.8%). Non-artemisinin therapy was stocked by 69.6% of the private sector and this was most commonly SP (57.6%). SP availability varied by outlet type, from 25.6% of private-for-profit facilities to 85.2% of general retailers.

**Table 5:** Availability of malaria commodities in the private sector

	Private For-Profit Facility	Registered pharmacy	Unregistered pharmacy	General retailer	Total private sector
	% (95% CI)	% (95% CI)	% (95% CI)	% (95% CI)	% (95% CI)
Availability of anti-malarials among all screened outlets	N = 360	N = 145	N = 493	N = 14,204	N = 15,202
Any anti-malarial	76.4	93.3	87.2	2.4	7.1
	(70.4, 81.4)	(87.8, 96.5)	(82.8, 90.6)	(1.7, 3.5)	(6.4, 7.9)
Availability of anti-malarials among anti-malarial-stocking outlets	N = 280	N = 134	N = 428	N = 244	N = 1086
QAACT	59.3	73.2	64.3	9.9	46.7
	(51.2, 67.0)	(65.6, 79.7)	(58.6, 69.7)	(5.1, 18.4)	(39.4, 54.1)
Non-QAACT	52.3	76.8	48.5	3.0	37.9
	(44.6, 59.9)	(68.7, 83.3)	(41.4, 55.6)	(1.3, 6.8)	(31.5, 44.7)
SP	25.6	49.9	55.2	85.2	57.6
	(19.5, 32.9)	(38.9, 61.0)	(47.1, 63.0)	(75.1, 91.6)	(50.8, 64.0)
Any non-artemisinin therapy	50.3	57.2	68.7	88.5	69.6
	(42.8, 57.8)	(45.1, 68.4)	(60.6, 75.8)	(78.9, 94.1)	(63.2, 75.4)
Artesunate injection	10.1	1.7	1.2	0.0	2.9
	(5.9, 16.8)	(0.5, 5.6)	(0.5, 2.6)	–	(1.7, 4.7)
Availability of blood testing among anti-malarial-stocking outlets^a^	N = 307	N = 138	N = 467	N = 310	N = 1222
Any diagnostic test	66.9	22.4	12.1	0.2	20.8
	(59.2, 73.8)	(16.4, 29.8)	(9.1, 16.0)	(0.0, 1.4)	(16.5, 25.8)
Malaria microscopy	48.1	8.8	3.2	0.0	12.4
	(39.1, 57.3)	(5.0, 15.1)	(2.0, 5.3)	–	(9.1, 16.7)
RDT	36.0	16.0	9.5	0.2	12.6
	(30.2, 42.3)	(11.1, 22.5)	(7.0, 13.0)	(0.0, 1.4)	(10.0, 15.8)

Among outlets stocking anti-malarials todays or within the past 3 months

The availability of diagnostic testing among anti-malarial-stocking private sector outlets was 20.8% (12.4% malaria microscopy and 12.4% RDT), and varied by outlet type: private for–profit facilities (66.9%), registered pharmacies (22.4%), unregistered pharmacies (12.1%) and general retailers (0.2%).

### Anti-malarial market share

**Fig. 1:**
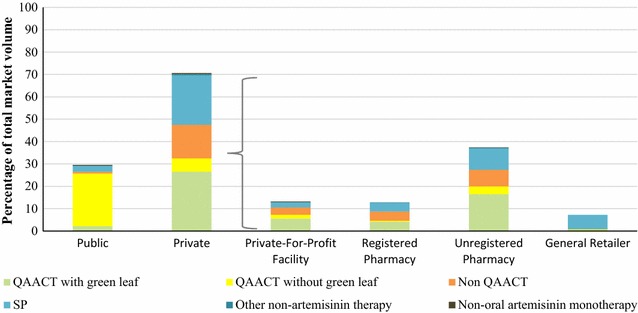
Anti-malarial market share, by outlet type and anti-malarial type

Figure 1 illustrates the anti-malarial market share (the amount of anti-malarials sold or distributed during the week preceding the survey) across the public and private sector, according to outlet type and by anti-malarial. In total, 70.6% of anti-malarials were distributed through the private sector. Most of the private sector distribution was through unregistered pharmacies (37.3%) followed by private for-profit health facilities (13.4%), registered pharmacies (12.8%) and general retailers (7.1%). Anti-malarial market share for the public sector was 29.4% and nearly all treatments distributed were QAACT medicines (25.6%). Within the private sector, 32.5% of the market share was accounted for by QAACT medicines, followed by non-QAACT medicines (15.0%) and non-artemisinin therapy (27.2%), which was typically SP (22.1%).

**Fig. 2:**
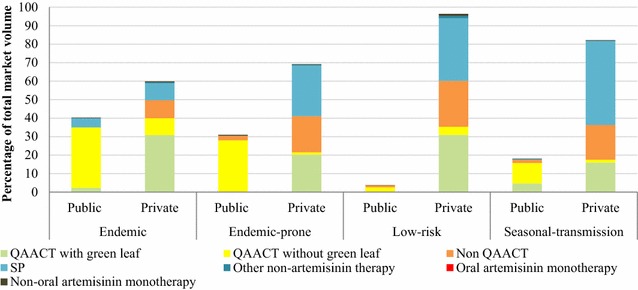
Anti-malarial market share, by epidemiological zones

The private-sector market share of all anti-malarials varied across epidemiological zones (Fig. 2): endemic areas (59.2%), endemic-prone areas (68.8%), seasonal-transmission areas (81.8%) and low-risk areas (94.9%). QAACT medicines market share was highest in endemic areas where (74.9% of anti-malarials distributed were QAACT), and lower in other zones: endemic-prone areas (49.4%), seasonal-transmission areas (32.2%) and low-risk areas (37.9%). Market share for non-QAACT medicines was lowest in endemic areas (9.7%) and highest in low-risk areas (24.8%). SP market share was 14.2% in endemic areas, 27.5% in endemic-prone areas, 45.7% in seasonal-transmission areas and 33.9% in low-risk areas. SP was exclusively distributed by the private sector in endemic-prone and low-risk areas, and mostly by the private sector in seasonal-transmission areas (0.3% of the public sector and 45.4% of the private sector).

### Malaria blood testing market share

**Fig. 3:**
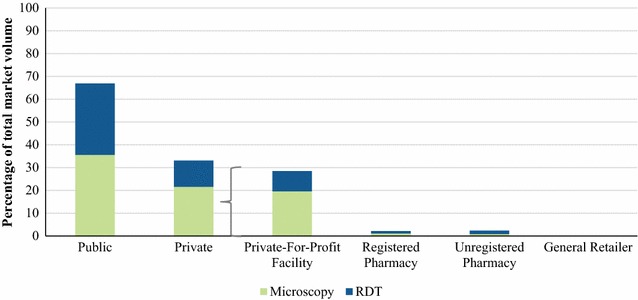
Malaria testing market share, by outlet type and type of test

Figure 3 shows that 66.9% of malaria blood testing was performed in the public sector and within the sector the market share of malaria microscopy (35.6%) and RDTs (31.3%) was comparable. A third (33.1%) of blood testing market share was delivered through private sector outlets and testing was more commonly performed through malaria microscopy (21.6%) than RDTs (11.5%). Notably, majority of the private sector malaria tests were delivered primarily by private-for-profit health facilities (28.5%) and was rare among registered pharmacies (2.2%), unregistered pharmacies (2.4%) and general retailers (0%).

### Private sector price for malaria treatments and blood testing

Table 6 summarizes the private sector median price for anti-malarials and blood testing. The median retail price for one QAACT AETD was $1.31 (IQR: 1.00–1.51). Non-QAACT AETD was $3.52 (IQR: 1.51–5.02). The median price for a SP AETD was $0.45 (IQR: 0.30–0.50) (Table 6).

**Table 6:** Median private sector price

	N	Median	Interquartile range
Median price of a tablet AETD			
QAACT	741	$1.31	1.00–1.51
Non-QAACT	845	$3.52	1.51–5.02
SP	768	$0.45	0.30–0.50
Median price of one test			
Malaria microscopy for an adult	204	$1.00	0.50–1.51
Malaria microscopy for a child	203	$1.00	0.50–100
Malaria RDT for an adult	179	$1.00	1.00–1.00
Malaria RDT for a child	178	$1.00	0.50–1.00

In regard to the cost of malaria blood testing, an adult patient was required to pay a median cost $1.00 to receive a malaria test using microscopy (IQR: 0.50–1.51) or RDT (IQR: 1.00–1.00). Similarly, the price of testing for children, either with microscopy or RDT, was $1.00 [microscopy (IQR: 0.50–100) or RDT (IQR: 0.50–100)].

### Provider perceptions of the most effective treatment for uncomplicated malaria

**Fig. 4:**
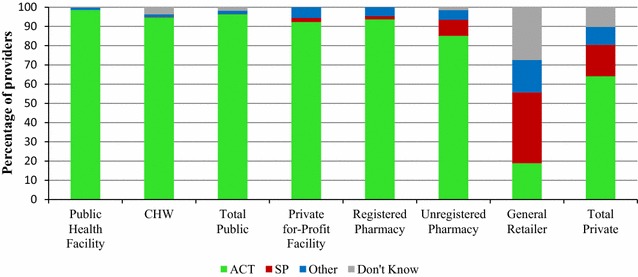
Provider perceptions on the most effective treatment for uncomplicated malaria in an adult

Figure 4 illustrates the extent to which providers perceived ACT as the most effective treatment for uncomplicated malaria in an adult. Nearly all providers (96.3%) in the public sector perceived ACT as the most effective treatment for uncomplicated malaria for adults. In the private sector, 64.1% perceived ACT to be the most effective, 16.4% perceived SP as the most effective, 9.2% cited another anti-malarial, and 10.2% did not know. General retailers and unregistered pharmacy providers were the most common outlet types to cite an anti-malarial other than an ACT or to report that they did not know what was the most effective treatment for malaria in an adult (81.1 and 14.8%, respectively). With regards to perceptions of the most effective anti-malarial for a child, a similar pattern was observed: 95.3% public sector cited an ACT, versus only 61.2% of private providers (Additional file 4).

## Discussion

This study provided a complete picture of the malaria testing and treatment landscape across public and private sectors in Kenya in 2016. Findings suggest that public sector readiness to test and appropriately treat malaria in Kenya is relatively high, but there is gap in availability of both testing and treatment that must be addressed in order to achieve universal coverage. In the private sector, where the majority of anti-malarials are distributed, the extent to which providers have malaria testing and appropriate anti-malarial medicines available is sub-optimal. These findings in combination with results from previous survey rounds, and current evidence from the different epidemiological zones, point to the progress which has been achieved as well as recommendations for improving malaria case management in Kenya.

### Public sector readiness to test and treat for malaria

Previous ACTwatch studies have demonstrated high availability of QAACTs and malaria blood testing since 2010 and 2014 respectively [16]. Nonetheless in 2016, one in five public health facilities did not have both QAACT and malaria blood testing available, pointing to the need to close this gap in order to achieve universal coverage. Furthermore, while availability of QAACT was generally high, the implementation of the AL policy includes delivery of four different AL pack sizes (6, 12, 18 and 24 tablets) suitable for management of four different weight categories of patients. Availability of the different weight categories was more variable among public health facilities, with less than 40% of outlets having the 18 tablet pack size in stock. Only half of the public health facilities stocked treatment for severe malaria, injectable artesunate, and this was similar to levels in 2014 [18]. SP for IPTp was available in 70% of public health facilities in endemic areas where it is recommended for IPTp, and rarely found among public health facilities outside of the endemic areas, illustrating alignment with national guidelines in these areas.

Some of the gaps found among public health facilities may be explained by changes in the supply and distribution of anti-malarial medicines and RDTs in the public sector. The Government of Kenya transitioned from a traditional central medical store using a push-distribution system to a pull-distribution system for malaria commodities, through the Kenya Medical Supplies Agency (KEMSA). However, the role of KEMSA to provide health commodities according to the requested needs (“pull” system) rather than allocated proportions of the total supply (“push” system) presented a number of challenges [16], and resulted in frequent stock-outs and erratic supplies of commodities to public health facilities. This has been attributed to limited capacity to quantify needs, insufficient budget, and challenges in maintaining a full pipeline of commodities to meet county specific needs [5]. As a means to prevent irrational anti-malarial medicines and RDT procurements and stock-outs within the public sector, more recently the National Malaria Control Programme and KEMSA have implemented a “smart push” system for malaria commodities. This strategy sets limits on the maximum quantity of malaria commodities that can be supplied to a facility, depending on the level of care and the epidemiological area. This strategy may help to further close the gap in availability of both testing and treatment in the public sector and will be important to safeguard the gains made nationally in malaria case management.

The reach of the public sector has been extended to the community-level through the training and equipping of CHW with malaria case management skills and supplies in endemic areas since 2012 [3]. The findings illustrate that a small portion of CHWs were ready for malaria case management and these were restricted to endemic and endemic-prone areas where interventions are being piloted. Low levels of QAACT and RDT availability even within these areas may be explained by the lack of a consistent public sector malaria commodity supply as previously discussed. Changes to the supply and distribution of anti-malarial medicines in the public sector has been described as contributing to delays in implementation of the malaria CHW strategy as public health facilities were either reluctant to give limited malaria commodities to CHWs or had no commodities to supply CHWs [5]. Maintaining a network of trained and equipped CHWs will be central to ensuring access to malaria case management services in rural areas. Key challenges to be addressed include gaps in CHW motivation and retention, training and maintaining supervision [17].

### The role of the private sector

Consistent with previous studies [18], the private sector plays an important role in malaria case management given almost three-fourths of all anti-malarials were distributed through this sector. While unregistered pharmacies comprised most of the market share, other private facilities also contributed to the anti-malarial distribution, including general retailers. As national policy stipulates that only private for-profit health facilities and registered pharmacies are licensed to sell medications, the results from this study suggest many anti-malarial medicines are being distributed through unregulated sources. Indeed, the Kenya Pharmacy and Poisons Board stipulates that for outlets to legally administer medicines, including anti-malarials, they must be registered with the board. In addition, medicines may only be dispensed under the supervision of a certified pharmacist or pharmaceutical technologists [19]. However, given the widespread distribution of anti-malarials through unregistered pharmacies and general retailers, strategies are needed to engage with these outlet types. One option may be to permit the licensing of unregistered pharmacies through accreditation programmes, or through partnerships with the public sector, as a means to increase access to appropriate malaria case management services. Evidence from other countries has demonstrated that the registration, training and supervision of the private sector, can lend to improvements in malaria commodity ACT availability, distribution and provider performance [20–23].

### Private sector readiness to test and treat for malaria

Among private sector outlets in the business of anti-malarial distribution, fewer than half had QAACT available in 2016, reflecting a decrease from levels reported in 2011 (60%) and 2014 (71%) [18]. Although the private sector CPM for QAACT administered by the Global Fund continued through 2016 in Kenya, important changes and challenges in the post-pilot period are likely to have contributed to the decline in availability. Since the AMFm, Kenya transitioned from a dedicated donor funding to a country specific grant funding mechanism, which was further amplified by a reduction in funding for co-paid ACTs. Indeed, the number of subsidized QAACT doses delivered to Kenya’s private sector in 2015 through the CPM was half of what it was in 2012 [9]. In this context, lower availability of QAACT in the private sector as compared to previous rounds can largely be explained by a more limited supply and availability of these anti-malarials given reduced funding.

Only one in five private providers stocking anti-malarial medicines had confirmatory testing available and this was highest among private for-profit facilities, reflecting national policy which only permits these types of facilities to administer confirmatory testing. While the private sector distributed the majority of anti-malarial medicines, only one-third of malaria blood tests were performed by private outlets, suggesting that presumptive treatment is common. Permitting other private sector outlet types to administer diagnostic testing may be one means to increase access to confirmatory testing as evidenced by a number of private sector RDT pilot initiatives [24–26]. Studies from Kenya have shown that registered pharmacies participating in a RDT pilot almost always correctly managed negative patients as per the government recommended treatment algorithm [11]. In addition, there were few differences in the performance of pharmacies and private for-profit health facilities, lending to the author’s conclusion that malaria diagnosis through RDTs can take place safely and effectively across a variety of channels. Given the role of unregistered pharmacies in Kenya, these facilities in particular may be critical to increasing access to confirmatory testing prior to treatment, though future research is merited to assess the feasibility of introducing RDTs among these outlet types. This is particularly pertinent as Kenya is deciding whether to legalize and/or subsidize RDTs in registered pharmacies as part of its malaria control strategy.

### Market share

Most of the anti-malarial market share comprised of QAACT, while one in four anti-malarials distributed were SP—and most commonly distributed through the private sector. Market share for QAACT was highest in endemic areas as compared with other epidemiological areas where distribution of SP was relatively common. In seasonal-transmission areas, SP accounted for nearly half of all anti-malarial distribution with nearly all of these treatments going through the private sector. SP accounted for about one-third of anti-malarials distributed in endemic-prone and low-risk areas. This finding suggests that SP is being used for malaria case management within the private sector and outside of endemic areas and points to the need to ensure continued availability of affordable QAACT in the private sector across the country, and not just focused in endemic areas. Furthermore, these findings suggest that unless effective private sector strategies are identified and implemented, further decline in private sector availability may be observed. In absence of suboptimal private sector engagement and support, and social and behaviour change communication for providers and consumers to raise awareness of QAACT, Kenya may see an increasing market share for SP.

Several reasons may explain the ongoing distribution of SP for case management. This may be in part driven by price as QAACT was approximately three times more expensive than SP in the private sector. While the price of SP has remained consistently low since 2010, the price of QAACT has fluctuated, initially dropping to nearly the same price as SP in 2011 but then increasing by 2014 [18]. The persistence of inexpensive SP may inhibit the uptake of QAACT even with a subsidy mechanism in place, and especially in the context of a reduced subsidy since the AMFm period. Furthermore, in the absence of supportive interventions to increase awareness of subsidized ACT (which ceased in 2015), providers and consumers may have less awareness of the subsidized ACT medicine. Indeed, the results also illustrate how some private sector providers perceived non-ACT medicines as the most effective treatment for uncomplicated malaria. Finally, while availability of QAACT was low in the private sector, the data also illustrate how there was variability according to different pack sizes, perhaps causing providers to ration ACTs. Other evidence has suggested that providers will administer other artemisinin therapies over ACT if supply is uncertain and availability is lower than non-recommended treatments [27]. Additional research to understand provider and consumer practices and demand for SP will be valuable to provide insights into the ongoing distribution.

Nearly one in five anti-malarials distributed were non-QAACTs. Non-QAACT was about 2.5 times more expensive than QAACT in the private sector. This raises the question of why consumers would pay more for non-QAACT when less expensive QAACT are available? Other ACTwatch research has shown that non-QAACT products were primarily distributed by pharmacies and drug stores in urban areas of Kenya. Thus the distribution of non-QAACT may reflect a higher purchasing power of urban consumers [28]. More research is needed to understand the reason for provider dispensing practices of non-QAACT as well as consumer preferences. Addressing the availability and distribution of non-QAACT will also require addressing elements of the supply chain, as well a product registration, manufacturing, importation laws and regulation.

### Limitations

General limitations of the study design which are applicable to all ACTwatch outlet surveys have been described in detail elsewhere [12, 14, 29]. In particular, the study is descriptive in nature and the results are unable to provide analytical information on the causal effects of interventions on the malaria testing and diagnostic landscape.

## Conclusions

Although public sector readiness to test and appropriately treat malaria in Kenya is relatively high, there is a gap in availability of both testing and treatment that must be addressed in order to achieve universal coverage. Also critical to achieving universal coverage with appropriate malaria case management in Kenya is the private sector, where the majority of anti-malarials are distributed. This includes distribution of nearly half of all anti-malarial medicines by unregistered and unregulated pharmacies and general retail outlets. There is need for strategies to effectively engage or regulate these outlet types. While private sector readiness to treat malaria according to national guidelines improved with a large scale QAACT subsidy piloted under the AMFm, availability of QAACT has subsequently declined to less than half of anti-malarial stocking private sector outlets in 2016. Given the continued role of the private sector in anti-malarial distribution, there is need for a renewed commitment and effective strategies to ensure access to affordable QAACT in the private sector. Additionally, given the aim of confirmatory testing prior to treatment, the low availability of testing particularly among the unregistered pharmacies, suggests need to examine policies and strategies for private sector malaria testing.

## Additional files

**Additional file 1.** Detailed sample description.

**Additional file 2.** Availability of QA AL, among all screened public sector outlets.

**Additional file 3.** Availability of QAACT among anti-malarial stocking outlets, by strata.

**Additional file 4.** Proportion of providers who report an ACT was the most effective anti-malarial medicine for a child.

## Abbreviations

AETD: adult equivalent treatment dose
ACT: artemisinin-based combination therapy
AMFm: Affordable Medicines Facility for malaria
AL: artemether–lumefantrine
CHW: community health worker
CPM: co-payment mechanism
IPTp: intermittent treatment as prevention during pregnancy
IQR: inter-quartile range
KEMSA: Kenya Medical Supplies Agency
SP: sulfadoxine–pyrimethamine
RDT: rapid diagnostic test
QAACT: quality-assured ACT

## References

[1] WHO. World Malaria Report. Geneva, Swizterland, World Health Organization; 2016. http://www.who.int/malaria/publications/world-malaria-report-2016/report/en/. Accessed 10 June 2017.

[2] Division of Malaria Control (2013). Kenya annual malaria report 2012/2013.

[3] National Malaria Control Programme (NMCP) and ICF International. Kenya Malaria Indicator Survey. 2015.

[4] Snow RW, Kibuchi E, Karuri SW, Sang G, Gitonga CW, Mwandawiro C (2015). Changing malaria prevalence on the Kenyan Coast since 1974: climate, drugs and vector control. PLoS ONE.

[5] President’s Malaria Initiative. Malaria Operational Plan FY 2016. 2016. https://www.pmi.gov/docs/default-source/default-document-library/malaria-operational-plans/fy16/fy-2016-kenya-malaria-operational-plan.pdf?sfvrsn=5. Accessed 24 Oct 2017.

[6] Amin AA, Zurovac D, Kangwana BB, Greenfield J, Otieno DN, Akhwale WS (2007). The challenges of changing national malaria drug policy to artemisinin-based combinations in Kenya. Malar J..

[7] Ministry of Health (2014). National guidelines for the diagnosis, treatment and prevention of malaria in Kenya.

[8] AMFm Independent Evaluation Team. Independent Evaluation of Phase 1 of the Affordable Medicines Facility-malaria (AMFm), Multi-Country Independent Evaluation Report: Final Report. Calverton, Maryland and London: ICF International and London School of Hygiene and Tropical Medicine. 2012.

[9] Tougher S, Hanson K, Goodman C, ACTwatch Group (2017). What happened to anti-malarial markets after the Affordable Medicines Facility-malaria pilot? Trends in ACT availability, price and market share from five African countries under continuation of the private sector co-payment mechanism. Malar J..

[10] Tougher S, Ye Y, Amuasi JH, Kourgueni IA, Thomson R, Goodman C, ACTwatch Group (2012). Effect of the Affordable Medicines Facility-malaria (AMFm) on the availability, price, and market share of quality-assured artemisinin-based combination therapies in seven countries: a before-and-after analysis of outlet survey data. Lancet.

[11] PSI. Transforming the Private Sector to Support Universal Malaria Diagnostic Coverage Lessons learned from Kenya, Madagascar and Tanzania. Washington, DC. 2017.

[12] Shewchuk T, O’Connell KA, Goodman C, Hanson K, Chapman S, Chavasse D (2011). The ACTwatch project: methods to describe anti-malarial markets in seven countries. Malar J..

[13] Kenya National Bureau of Statistics (2010). 2009 Kenya population and housing census.

[14] O’Connell KA, Gatakaa H, Poyer S, Njogu J, Evance I, Munroe E (2011). Got ACTs? Availability, price, market share and provider knowledge of anti-malarial medicines in public and private sector outlets in six malaria-endemic countries. Malar J..

[15] WHO. Guidelines for the treatment of malaria. Third edition. Geneva, Switzerland, World Health Organization; 2015. http://www.who.int/malaria/publications/atoz/9789241549127/en/. Accessed June 1 2017.

[16] Hanson K, Goodman C (2017). Testing times: trends in availability, price, and market share of malaria diagnostics in the public and private healthcare sector across eight sub-Saharan African countries from 2009 to 2015. Malar J..

[17] Aronovich D, Kinzett S (2001). Assessment of the health commodity supply chains and the role of KEMSA.

[18] ACTwatch Group. ACTwatch Study Reference Document: Kenya Outlet Survey 2014 Washington, DC. 2014.

[19] Rusk A, Smith N, Menya D, Obala A, Simiyu C, Khwa-Otsyula B (2012). Does anti-malarial drug knowledge predict anti-malarial dispensing practice in drug outlets? A survey of medicine retailers in western Kenya. Malar J..

[20] Rutta E, Liana J, Embrey M, Johnson K, Kimatta S, Valimba R (2015). Accrediting retail drug shops to strengthen Tanzania’s public health system: an ADDO case study. J Pharm Policy Pract..

[21] Phok S, Lek D, ACTwatch Group (2017). Evidence on anti-malarial and diagnostic markets in Cambodia to guide malaria elimination strategies and policies. Malar J..

[22] Phanalasy S, ACTwatch Group (2017). The malaria testing and treatment landscape in the southern Lao People’s Democratic Republic (PDR). Malar J..

[23] Michael D, Mkunde SP (2017). The malaria testing and treatment landscape in mainland Tanzania, 2016. Malar J..

[24] Cohen J, Fink G, Berg K, Aber F, Jordan M, Maloney K (2012). Feasibility of distributing rapid diagnostic tests for malaria in the retail sector: evidence from an implementation study in Uganda. PLoS ONE.

[25] Yeung S, Patouillard E, Allen H, Socheat D (2011). Socially-marketed rapid diagnostic tests and ACT in the private sector: 10 years of experience in Cambodia. Malar J..

[26] Mbonye AK, Ndyomugyenyi R, Turinde A, Magnussen P, Clarke S, Chandler C (2010). The feasibility of introducing rapid diagnostic tests for malaria in drug shops in Uganda. Malar J..

[27] Rao VB, Schellenberg D, Ghani AC (2013). Overcoming health systems barriers to successful malaria treatment. Trends Parasitol..

[28] Newton PN, Hanson K, Goodman C, ACTwatch Group (2017). Do anti-malarials in Africa meet quality standards? The market penetration of non quality-assured artemisinin combination therapy in eight African countries. Malar J..

[29] O’Connell KA, Poyer S, Solomon T, Munroe E, Patouillard E, Njogu J (2013). Methods for implementing a medicine outlet survey: lessons from the anti-malarial market. Malar J.

