# Supplementary material for: The malaria testing and treatment landscape in Kenya: results from a nationally representative survey among the public and private sector in 2016
Source: Malar J. 2017 Dec 21;16:494. doi: 10.1186/s12936-017-2089-0 (PMC5740898; doi:10.1186/s12936-017-2089-0)
Supplement: Supplementary file 1 — Additional file 1. Detailed sample description. [file 12936_2017_2089_MOESM1_ESM.docx]

### Additional File 1: Detailed sample description

|  | Public  Health  Facility | Community Health Worker | Private Not For-Profit  Facility | Private  For-Profit  Facility | Registered Pharmacy | Unregistered Pharmacy | General Retailer | **TOTAL** |
| --- | --- | --- | --- | --- | --- | --- | --- | --- |
| **Number of outlets screened** | 796 | 1,815 | 39 | 360 | 145 | 493 | 14,204 | 17,852 |
| Census | 197 | 1,815 | 39 | 360 | 145 | 493 | 14,204 | 17,253 |
| Booster | 599 | 0 | 0 | 0 | 0 | 0 | 0 | 599 |
| **Number of outlets eligible and interviewed** | 775 | 217 | 35 | 329 | 138 | 467 | 310 | 2,271 |
| Census | 190 | 217 | 35 | 329 | 138 | 467 | 310 | 1,686 |
| Booster | 585 | 0 | 0 | 0 | 0 | 0 | 0 | 585 |
| **Number of outlets eligible but not interviewed** | 1 | 0 | 1 | 4 | 3 | 10 | 1 | 20 |
| Census | 1 | 0 | 1 | 4 | 3 | 10 | 1 | 20 |
| Booster | 0 | 0 | 0 | 0 | 0 | 0 | 0 | 0 |
| **Number of interviewed outlets with at least one anti-malarial in stock on the day of the survey** | 734 | 65 | 32 | 280 | 134 | 428 | 244 | 1,917 |
| Census | 179 | 65 | 32 | 280 | 134 | 428 | 244 | 1,362 |
| Booster | 555 | 0 | 0 | 0 | 0 | 0 | 0 | 555 |
| **Number of interviewed outlets with at least one anti-malarial in stock on the day of the survey or at least one anti-malarial reportedly in stock in the previous 3 months** | 767 | 187 | 34 | 307 | 138 | 467 | 310 | 2,210 |
| Census | 190 | 187 | 34 | 307 | 138 | 467 | 310 | 1,633 |
| Booster | 577 | 0 | 0 | 0 | 0 | 0 | 0 | 577 |
| **Number of interviewed outlets that provide malaria blood testing, but do not stock anti-malarial medicines** | 32 | 55 | 2 | 35 | 0 | 2 | 0 | 126 |
| Census | 6 | 55 | 2 | 35 | 0 | 2 | 0 | 100 |
| Booster | 26 | 0 | 0 | 0 | 0 | 0 | 0 | 26 |
| **Number of interviewed outlets that reported distributing anti-malarials in the week prior to the survey** | 547 | 44 | 17 | 229 | 114 | 384 | 167 | 1,502 |
| Census | 132 | 44 | 17 | 229 | 114 | 384 | 167 | 1,087 |
| Booster | 415 | 0 | 0 | 0 | 0 | 0 | 0 | 415 |
| **Number of interviewed outlets that reported providing/distributing a malaria diagnostic test in the week prior to the survey** | 567 | 76 | 22 | 202 | 23 | 45 | 1 | 936 |
| Census | 136 | 76 | 22 | 202 | 23 | 45 | 1 | 505 |
| Booster | 431 | 0 | 0 | 0 | 0 | 0 | 0 | 431 |
