# Supplementary material for: The malaria testing and treatment landscape in Kenya: results from a nationally representative survey among the public and private sector in 2016
Source: Malar J. 2017 Dec 21;16:494. doi: 10.1186/s12936-017-2089-0 (PMC5740898; doi:10.1186/s12936-017-2089-0)
Supplement: Supplementary file 2 — Additional file 2. Availability of QA AL, among all screened public sector outlets. [file 12936_2017_2089_MOESM2_ESM.docx]

### Additional File 2: Availability of QA AL, among all screened public sector outlets

|  | **Public**  **Health Facility** | **Community Health Worker** |
| --- | --- | --- |
|  | %  (95% CI) | %  (95% CI) |
|  | N=796 | N=1,815 |
| AL 20/120, 6 tablets | 66.4 | 1.8 |
|  | (59.1, 73.0) | (0.6, 4.8) |
| AL 20/120 , 12 tablets | 63.3 | 1.6 |
|  | (56.6, 69.6) | (0.5, 5.1) |
| AL 20/120, 18 tablets | 37.0 | 0.2 |
|  | (29.8, 44.9) | (0.1, 0.8) |
| AL 20/120, 24 tablets | 72.7 | 1.3 |
|  | (67.3, 77.5) | (0.4, 4.3) |

*Inclusive of 39 public-not-for profit facilities
