# Supplementary material for: The malaria testing and treatment landscape in Kenya: results from a nationally representative survey among the public and private sector in 2016
Source: Malar J. 2017 Dec 21;16:494. doi: 10.1186/s12936-017-2089-0 (PMC5740898; doi:10.1186/s12936-017-2089-0)
Supplement: Supplementary file 3 — Additional file 3. Availability of QAACT among anti-malarial stocking outlets, by strata. [file 12936_2017_2089_MOESM3_ESM.docx]

### Additional File 3: Availability of QAACT among anti-malarial stocking outlets, by strata

|  | | **Private**  **For-Profit Facility** | | | **Registered Pharmacy** | | | **Unregistered Pharmacy** | | | **General Retailer** | | | | **Total**  **Private Sector** | | | | |
| --- | --- | --- | --- | --- | --- | --- | --- | --- | --- | --- | --- | --- | --- | --- | --- | --- | --- | --- | --- |
|  | | %  (95% CI) | | | %  (95% CI) | | | %  (95% CI) | | | %  (95% CI) | | | | %  (95% CI) | | | | |
|  | | Endemic N=54  Highland N=57  Low risk N=118  Seasonal low N=51 | | | Endemic N=20  Highland N=21  Low risk N=83  Seasonal low N=10 | | | Endemic N=134  Highland N=68  Low risk N=172  Seasonal low N=54 | | | Endemic N=48  Highland N=26  Low risk N=42  Seasonal low N=128 | | | | Endemic N=256  Highland N=172  Low risk N=415  Seasonal low N=243 | | | | |
|  | |  | | |  | | |  | | |  | | | |  | | | | |
| Endemic | | 70.5 | | | 77.8 | | | 75.1 | | | 42.3 | | | | 66.7 | | | | |
|  | | (57.8, 80.6) | | | (54.0, 91.2) | | | (66.6, 82.0) | | | (20.4, 67.7) | | | | (56.6, 75.5) | | | | |
|  | |  | | |  | | |  | | |  | | | |  | | | | |
| Highland | | 57.3 | | | 61.9 | | | 45.6 | | | 6.9 | | | | 43.0 | | | | |
|  | | (42.3, 71.1) | | | (46.2, 75.5) | | | (27.2, 65.4) | | | (1.2, 30.1) | | | | (32.0, 54.7) | | | | |
|  | | | | | | | | | | | |  | | | |  |  |  |  |
|  | (42.3, 71.1) | | (46.2, 75.5) | | | (27.2, 65.4) | | | (1.2, 30.1) | | | | (32.0, 54.7) | | | | | | |
| Seasonal low | 72.4 | | 80.4 | | | 71.5 | | | 3.4 | | | | 33.8 | | | | | | |
|  | (56.2, 84.3) | | (41.7, 95.9) | | | (52.5, 85.0) | | | (1.2, 9.2) | | | | (23.6, 45.8) | | | | | | |
| Low risk | 48.4 | | | 73.3 | | | 56.3 | | | 0.0 | | | | 42.2 | | | | | |
|  | (31.1, 66.2) | | | (63.0, 81.5) | | | (48.8, 63.5) | | | - | | | | (26.8, 59.2) | | | | | |
|  |  | |  | | |  | | |  | | | |  | | | | | | |
