# Supplementary material for: The malaria testing and treatment landscape in Kenya: results from a nationally representative survey among the public and private sector in 2016
Source: Malar J. 2017 Dec 21;16:494. doi: 10.1186/s12936-017-2089-0 (PMC5740898; doi:10.1186/s12936-017-2089-0)
Supplement: Supplementary file 4 — Additional file 4. Proportion of providers who report an ACT was the most effective anti-malarial medicine for a child. [file 12936_2017_2089_MOESM4_ESM.docx]

Additional File 4: Proportion of providers who report an ACT was the most effective anti-malarial medicine for a child

| Public  Health  Facility | Community Health Worker | Total  Public  Sector | Private  For-Profit  Facility | Registered Pharmacy | Unregistered Pharmacy | General Retailer | Total Private  Sector |
| --- | --- | --- | --- | --- | --- | --- | --- |
| %  (95% CI) | %  (95% CI) | %  (95% CI) | %  (95% CI) | %  (95% CI) | %  (95% CI) | %  (95% CI) | %  (95% CI) |
| N=767 | N=187 | N=988 | N=307 | N=138 | N=467 | N=310 | N=1,222 |
| 97.8 | 92.7 | 95.3 | 88.1 | 94.1 | 84.0 | 15.1 | 61.2 |
| (96.2, 98.7) | (81.2, 97.4) | (90.7, 97.7) | (83.3, 91.7) | (87.4, 97.3) | (77.9, 88.7) | (9.3, 23.5) | (51.4, 70.2) |

*Inclusive of 34 public not-for profit health facilities
